# Longitudinal study of mental abilities in teaching primary school students with intellectual disabilities

**DOI:** 10.1192/j.eurpsy.2025.1185

**Published:** 2025-08-26

**Authors:** Y. Sidneva, A. Zakrepina, T. Butusova, L. Pronina

**Affiliations:** 1 rehabilitation, Clinical and Research Institute of Emergency Pediatric Surgery and Traumatology (CIEPST); 2 neuropsychiatry, National Medical Research Center of Neurosurgery named after N.N. Burdenko; 3 defectology, Federal State Budgetary Scientific Institution Institute of Correctional Pedagogics /FGBNU ICP, Moscow, Russian Federation

## Abstract

**Introduction:**

The results of achievements in mastering the program material of students of a special school by the end of the fourth year of study are characterized by a large variability in training, which allows us to assert the need to develop and apply special teaching techniques and methods, harmonize the academic workload

**Objectives:**

to study the mental capabilities and identify learning difficulties of fourth-grade students with mental disabilities in elementary school by the end of study

**Methods:**

Two samples of fourth-graders (132 students) were identified: Group 1 - 100 students with mild mental retardation, Group 2 - 32 students with severe intellectual disabilities. Methods: observation, pedagogical experiment, cluster analysis

**Results:**

Clustering criteria: volume and level of assimilation of program material in academic subjects; achievement of subject and personal results.

In group 1 (75%), 3 clusters were identified by academic performance in the main academic subjects, which are uniform in terms of the variability of dynamics, which indicates the homogeneity of the group of students; however, the groups differ in the level of achievement of subject and personal results (sufficient, average, minimal).

In group 2 (25%), 3 clusters were also identified, which are variable, but do not duplicate each other, and have an individual spread of indicators in the dynamics of subject and personal results within each cluster, which indicates the heterogeneity of the group of students in each cluster

**Conclusions:**

three levels of dynamics in the education of students with varying degrees of mental retardation were identified. The range in the variations of the results of the 2nd group of students with severe intellectual disabilities indicates the need for individualization of education, selection of partial programs taking into account the special educational needs of each student, and the development of special personalized approaches to education. Apparently, it is also necessary to select the optimal amount of academic workload and the level of complexity of the material studied, compliance with health-saving technologies in accordance with the individual psychological characteristics of the student

**Disclosure of Interest:**

None Declared

